# Inflammasome expression patterns during bone regeneration: predictive biomarkers and potential therapeutic targets in non-union formation

**DOI:** 10.3389/fimmu.2026.1903147

**Published:** 2026-09-16

**Authors:** Maximilian M. Menger, Oguzhan Nakisli, Benedikt J. Braun, Steven C. Herath, Daniel A.K. Schäfer, Dominik Saul, Michael D. Menger, Emmanuel Ampofo, Selina Wrublewsky, Matthias W. Laschke, Tina Histing

**Affiliations:** 1 Department of Trauma and Reconstructive Surgery, BG Clinic Tuebingen, Eberhard Karls University Tuebingen, Tuebingen, Germany; 2 Institute for Clinical and Experimental Surgery, Saarland University, PharmaScienceHub (PSH), Homburg, Germany; 3 Robert Bosch Center for Tumor Diseases (RBCT), Stuttgart, Germany

**Keywords:** AIM2, bone regeneration, fracture healing, inflammasome, inflammation, inflammatory cells, NLRP3, non-union

## Abstract

**Background:**

Non-union formation is one of the major complications in trauma and orthopedic surgery. The inflammatory response at the fracture site is crucial for determining the healing outcome. However, there is limited information on the expression profiles of inflammasomes during both successful fracture repair and non-union formation.

**Methods:**

Hence, we analyzed the cellular inflammatory response as well as inflammasome expression during successful bone regeneration and fracture healing failure in a standardized murine fracture and non-union model over 70 days. Bone healing was analyzed by means of X-ray, biomechanics, micro-computed tomography (µCT), histology as well as immunohistochemistry and Western blotting.

**Results:**

The analysis of the callus tissue in non-unions showed a slightly increased number of MPO-positive cells and a significantly increased number of macrophages during the early healing phase. The number of macrophages remained significantly elevated until 70 days after surgery, indicating an overshooting and prolonged inflammatory response when compared to unions. This was associated with an enhanced number of p16-positive cells. Additional Western blot analyses revealed distinct differences in the inflammasome expression profiles of unions and non-unions. In particular, non-unions presented with a higher expression of the nucleotide-binding oligomerization domain (NOD)-like receptor protein (NLRP)3 at 10 days. Furthermore, at 14 days non-unions showed a higher expression of NLRP1 whereas the expression of absent in melanoma (AIM)2 was significantly reduced.

**Conclusion:**

In a clinical setting, these distinct expression profiles may serve as predictive markers for fracture healing outcome. Moreover, in the future novel treatment approaches modulating inflammasome expression may be used to stimulate bone regeneration and eventually prevent non-union formation.

## Introduction

Delayed fracture healing and non-union formation belong to the major complications in trauma and orthopedic surgery (1). Bone healing failure not only results in significant pain and physical impairment for the patient, but is also associated with a prolonged and often unsuccessful rehabilitation, placing a considerable financial burden on the healthcare system (2, 3). During the last decades bone regeneration has been extensively studied in preclinical and clinical research. However, despite growing knowledge about the physiology of fracture repair, the cause for non-union formation remains unclear in a considerable number of cases (4).

Successful fracture healing is a highly complex, temporally and spatially coordinated process involving a plethora of cell types and growth factors (5). Notably, the inflammatory response following the injury is considered to be of particular importance for the healing outcome. Pro-inflammatory cytokines and cells stimulate the recruitment of mesenchymal stem cells (MSCs) to the fracture site. These MSCs provide a cell pool for the subsequent differentiation towards pro-angiogenic and pro-osteogenic cell lineages, initiating callus formation and bone regeneration (5–7). An overshooting or chronic inflammation, however, may have detrimental effects on the healing outcome (8). In the clinical setting, a prolonged inflammatory condition can be typically observed in different patient collectives, including aged or polytraumatized patients as well as individuals suffering from diabetes mellitus (7–9). Elucidating the inflammatory dysregulation and its impact on fracture repair may thus help to develop effective therapeutic approaches for the stimulation of bone regeneration.

In recent years, inflammasomes, i.e. large multimolecular protein complexes, have gained increasing attention. Among the most common and well-defined inflammasomes are nucleotide-binding oligomerization domain (NOD)-like receptor protein (NLRP)1 and NLRP3, NLR family CARD domain-containing protein (NLRC)4 as well as absent in melanoma (AIM)2 (10, 11). These inflammasomes play a vital role in the inflammatory response by promoting interleukin (IL)-1β/IL-18 maturation and cell pyroptosis (10). For inflammasome activation the apoptosis-associated speck-like protein containing a CARD (ASC) is recruited to the inflammasome. The ASC and the inflammasome activate pro-caspase-1 via CARD-CARD interaction. Subsequently, caspase-1 induces the maturation of IL-1β, IL-18 and gasdermin D (10).

Interestingly, accumulating evidence suggests that inflammasome complexes are not only central regulators of innate immunity, but are important mediators in bone maturation (12, 13), immunology and diseases, such as osteonecrosis and gouty arthritis (10). However, their expression profiles during bone regeneration and potential contribution to non-union formation remain elusive. Hence, the aim of the present study was to characterize the inflammasome expression and potential inflammatory dysregulation in non-unions compared to unions by means of two well-established murine bone healing models.

## Materials and methods

### Animals and experimental protocol

A total number of 70 adult CD-1 wildtype mice of both sexes (37 male and 33 female mice) were used in the present study. Of note male and female mice were equally distributed among the study groups to avoid possible effects of sex on bone regeneration and inflammation. The animals were bred at the Institute for Clinical and Experimental Surgery (Saarland University, Homburg, Germany) and housed on wood chips as bedding under standard laboratory conditions (temperature: 22 ± 2 °C; humidity: 55 ± 10%) on a standard 12-hour day/night cycle with free access to water and pellet chow (Altromin, Lage, Germany). For radiological, histological and immunohistochemical analyses a total of 40 animals were sacrificed at 7, 10, 14 and 70 days after surgery (n = 5 per group and time point). An additional 30 animals were sacrificed at 7, 10 and 14 days after surgery (n = 5 per group and time point) for Western blotting. The anesthetized animals were euthanized by cervical dislocation, and the femora were explanted and processed for further analyses. Notably, group allocation of the study animals was randomized and outcome assessors performed blinded analysis.

All experiments were performed according to the German legislation on the protection of animals and the National Institutes of Health Guide for the Care and Use of Laboratory Animals. The experiments were approved by the local authorities (State Office for Consumer Protection, Saarbrücken, Germany).

### Surgical procedures

To induce physiological bone regeneration (union group), a standardized femoral fracture model was used, as previously described in detail (14). For this purpose, the animals were anesthetized by ketamine (110 mg/kg body weight (BW)) and xylazine (12 mg/kg BW). Under sterile conditions, a 4 mm parapatellar incision was made at the medial aspect of the right knee. The patella was dislocated laterally, and a 0.5 mm hole was drilled into the intercondylar notch to access the intramedullary canal. Using a 0.4 mm injection needle, the greater trochanter was penetrated retrogradely. A 0.2 mm tungsten guide wire was then advanced into the intramedullary canal through the needle. After removal of the needle, a standardized transverse midshaft fracture of the right femur was created using a blunt guillotine device. Subsequently, an intramedullary medical-grade stainless-steel screw (MouseScrew^®^; RISystem AG, Landquart, Switzerland) was inserted over the pre-positioned guide wire to stabilize the fracture. The wound was closed with 6–0 synthetic sutures. For analgesia animals were treated with tramadol (1mg/ml) within the drinking water from 1 day preoperatively to 3 days postoperatively. Moreover, the animals received carprofen (5 mg/kg BW) during surgery. Appropriate fracture configuration and implant positioning were confirmed by digital radiography (MX-20; Faxitron X-ray Corporation, Wheeling, IL, USA).

To induce non-union formation (non-union group) a previously established pin-clip model was used (15). For this purpose, the animals were anesthetized by ketamine (110 mg/kg BW) and xylazine (12 mg/kg BW). Under sterile conditions, a 4 mm medial parapatellar incision was performed at the right knee and the patella was dislocated laterally. After drilling a 0.5 mm hole into the intercondylar notch, a 24G needle was implanted intramedullary and the wound was closed. To prevent secondary pin loss, the pin was flattened at the distal end. Subsequently, the middle of the femur was exposed through a lateral approach, and a custom-made clip was implanted ventro-dorsally into the femur. Thereafter, an osteotomy with a gap size of 1.8 mm was created under the metallic clip using a size-standardized spherical trephine. To avoid heat necrosis, drilling was performed under continuous saline cooling. The gap size was controlled by custom-made templates of 1.8 mm. The metallic clip inserted before gap creation guaranteed that the gap size could be maintained. The wound was closed with 6–0 synthetic sutures. Finally, the osteotomy and implant positioning were evaluated by digital radiography (MX-20; Faxitron X-ray Corporation).

### X-ray

To monitor the process of bone regeneration and identify possible implant dislocation, lateral radiographs were performed at 7, 10, 14 and 70 days after surgery before harvesting of the femora (MX-20; Faxitron X-ray Corporation). Moreover, bone regeneration was evaluated using the Goldberg score, with stage 0 indicating radiological non-union, stage 1 indicating possible union and stage 2 indicating radiological union (16).

### Biomechanics

After removing the soft tissue, the bending stiffness of the harvested femora was measured by a non-destructive approach using a 3-point-bending device (17). Loading was stopped individually when the load-displacement curve deviated > 1% from linearity. The bending stiffness was then calculated from the load-displacement diagram and is given as absolute bending stiffness (N/mm).

### Micro-computed tomography (µCT)

After biomechanical testing, the harvested femora were scanned (Skyscan 1176; Bruker, Billerica, MA, USA) at a spatial resolution of 9 μm with a standardized setup (tube voltage: 50 kV; current: 200 μA; intervals: 0.4°; exposure time: 3500 ms; filter: 0.5 mm aluminum). Images were stored in 3-dimensional arrays. To express gray values as mineral content (bone mineral density (BMD)), calcium hydroxyapatite (CaHA) phantom rods with known BMD values (0.250 g and 0.750 g CaHA/cm^3^) were used for calibration. The region of interest (ROI) defining the newly formed callus tissue was contoured manually excluding original cortical bone. The thresholding allowed the differentiation between poorly and highly mineralized bone. The thresholds to distinguish between poorly and highly mineralized bone were based upon visual inspection of the images, qualitative comparison with histological sections and other studies investigating bone repair and callus tissue by μCT (18, 19). A BMD of more than 0.642 g/cm^3^, resulting in gray values of 98-255, was defined as highly mineralized bone. A BMD value between 0.410 and 0.642 g/cm^3^, resulting in gray values of 68-97, was defined as poorly mineralized bone.

The following parameters were calculated from the callus ROI for each specimen: poorly and highly mineralized bone volume (BV) (mm³), the fraction of poorly and highly mineralized BV to tissue volume (TV) (%), trabecular thickness (mm), trabecular separation (mm) and trabecular number (1/mm).

### Histology and histomorphometry

Following the µCT scan, femora were fixed in 4% phosphate-buffered formalin for 24 h, decalcified in 10% ethylene-diamine-tetra-acetic acid (EDTA) solution for 2 weeks and then embedded in paraffin. Longitudinal sections through the femoral axis were cut and stained with Safranin-O for histomorphometric analyses. At a magnification of 12.5x (Olympus BX60 Microscope; Olympus, Shinjuku, Japan and Zeiss Axio Cam with Axio Vision 3.1; Zeiss, Oberkochen, Germany) structural indices were calculated according to the recommendations of Gerstenfeld et al. (20). The following histomorphometric parameters of the callus were evaluated: total callus area (mm^2^), the fraction of osseous tissue (%), cartilaginous tissue (%) and fibrous tissue (%). Each area was marked and calculated using the ImageJ analysis software (NIH, Bethesda, USA). Moreover, high-magnification images (200x magnification) were obtained for a better visualization of the callus tissue.

### Immunohistochemistry

To further analyze the cellular composition of the callus tissue, additional longitudinal tissue sections were cut. For the detection of inflammatory cells i.e. macrophages and granulocytes, the sections were stained using a rabbit anti-mouse CD68-antibody (1:300; Abcam, Cambridge, United Kingdom) and a rabbit anti-mouse myeloperoxidase (MPO)-antibody (1:100; Abcam). To detect p16-positive cells a rabbit anti-mouse p16-antibody (1:100; Merck, Darmstadt, Germany) was used. A goat anti-rabbit IgG-biotin antibody served as secondary antibody (Abcam).

Positive-stained cells within the callus tissue were analyzed at 400x magnification in a standardized manner. One high-power field (HPF) was placed in the central region of the callus (former fracture or osteotomy gap), while three to five additional HPFs were placed at each site within the periosteal region of the callus. The number of positive-stained cells within each HPF was counted and the mean for each specimen was determined.

### Western blot

Western blotting was performed to analyze the expression of different inflammasomes and cytokines in the callus tissue at 7, 10 and 14 days after surgery (n = 5 per group). These analyses included the inflammasomes NLRP1 (1:30; Santa Cruz, Dallas, TX, USA), NLRP3 (1:30; Cell Signaling Technology, Danvers, MA, USA), NLRC4 (1:30; Abcam) and AIM2 (1:30; Cell Signaling Technology) as well as the inflammatory cytokines IL-1β (1:30; Cell Signaling Technology) and IL-18 (1:30; Abcam).

Primary antibodies were followed by their corresponding horseradish peroxidase-conjugated secondary antibodies. Protein expression was visualized by means of luminol-enhanced chemiluminescence (ECL) after exposure of the membranes in an Intas ECL Chemocam Imager (Intas Science Imaging Instrument GmbH, Göttingen, Germany) and normalized to β-actin signals to correct for unequal loading. Data are given as pixel intensity*10^4^. Of note, the membranes were cut according to the molecular weight ranges of the target proteins prior to antibody incubation. The same membranes were used for NLRP1 and IL-1β at days 7, 10 and 14; NLRP3, AIM2 and IL-18 at day 7; and NLRC4, AIM2 and IL-18 at days 10 and 14. Consequently, the corresponding proteins share the same β-actin loading controls at these time points, resulting in the repeated presentation of the respective β-actin bands. Additional descriptive analyses of the ratios of mean inflammasome expression levels between unions and non-unions were calculated. The following ratios are demonstrated: NLRP3/NLRP1, NLRP3/AIM2, NLRP3/NLRC4, NLRC4/NLRP1, NLRC4/AIM2 and NLRP1/AIM2. Of note, the ratios should illustrate distinct expression signatures with potential additive or synergistic trends in inflammasome expression rather than to define a quantitative inflammasome ratio. Thus, the presented ratios should only be considered as densitometric indices.

### Statistical analysis

In all comparative analyses data are given as means ± standard error of the mean (SEM). Due to the small number of animals per group, normality testing (Kolmogorov-Smirnov) is underpowered. Therefore, comparisons between the two groups (union vs. non-union) were performed by applying the non-parametrical Mann-Whitney U test. Because the primary endpoint of our study was to compare unions vs. non-unions, statistical comparisons between the individual time points were not performed.

All statistics were performed using the SigmaPlot 13.0 software (Jandel Corporation, San Rafael, CA, USA) and GraphPad Prism software version 8.0 (GraphPad Software, La Jolla, CA, USA). A p‐value of < 0.05 was considered to indicate significant differences.

## Results

### X-ray

X-rays of the femora in the union group showed typical signs of endochondral fracture healing and subsequent callus remodeling (**Figure 1a**). In the non-union group, no signs of callus formation were evident, with a persisting bone defect at 70 days after surgery (**Figure 1b**). Notably, after 70 days the bone ends at the former osteotomy site were rounded, indicating the manifestation of atrophic non-union formation (**Figure 1b**).

**Figure 1:**
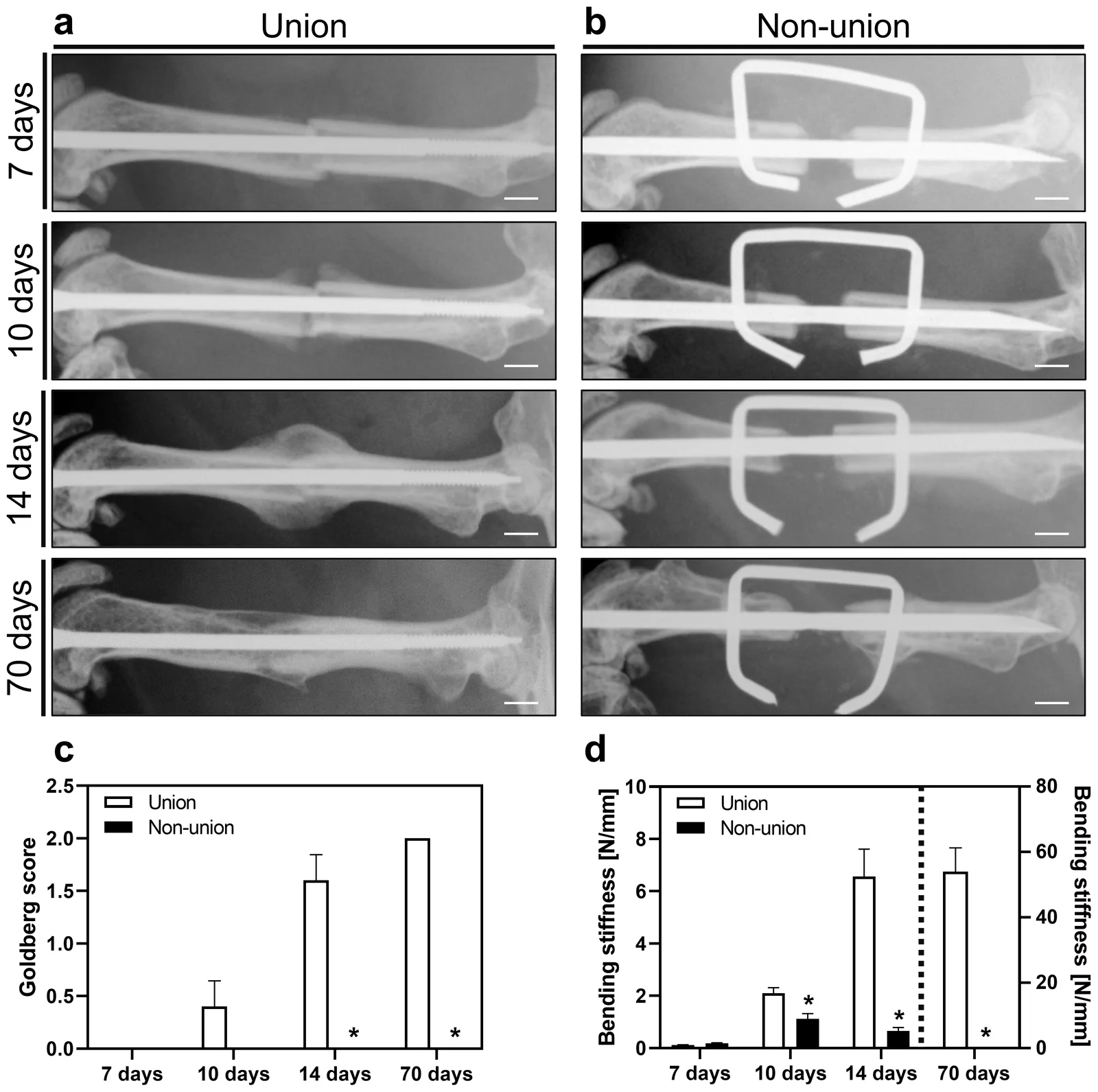
X-ray and biomechanics. **(a, b)** Representative X-rays of femora in the union **(a)** and non-union group **(b)** at 7, 10, 14 and 70 days after surgery. Scale bars: 1 mm. **(c)** X-ray analysis with the Goldberg score of the femora in the union and non-union group at 7, 10, 14 and 70 days after surgery (n = 5 per group and time point). **(d)** Biomechanical analysis of the bending stiffness (N/mm) of the femora in the union and non-union group at 7, 10, 14 and 70 days after surgery (n = 5 per group and time point). Bars to the left of the dotted line correspond to the left y-axis, whereas bars to the right of the dotted line correspond to the right y-axis. Data are given as mean ± SEM; *p < 0.05 vs. union (Mann-Whitney U test).

The quantitative radiographic analysis revealed a significantly lower Goldberg score of the femora at 14 and 70 days after surgery in the non-union when compared to the union group, demonstrating the lack of healing progression (**Figure 1c**).

### Biomechanics

The biomechanical analysis showed a significantly lower bending stiffness of the femora in the non-union group when compared to the union group at 10, 14 and 70 days after surgery (**Figure 1d**).

### µCT

In line with the X-ray analysis, additional radiological analyses of the explanted femora by µCT revealed successful bone regeneration during the entire observation period in the union group (**Figure 2a**). In contrast, the non-union group exhibited no signs of significant bone formation, with rounded and sclerosed bone ends at 70 days after surgery (**Figure 2b**).

**Figure 2:**
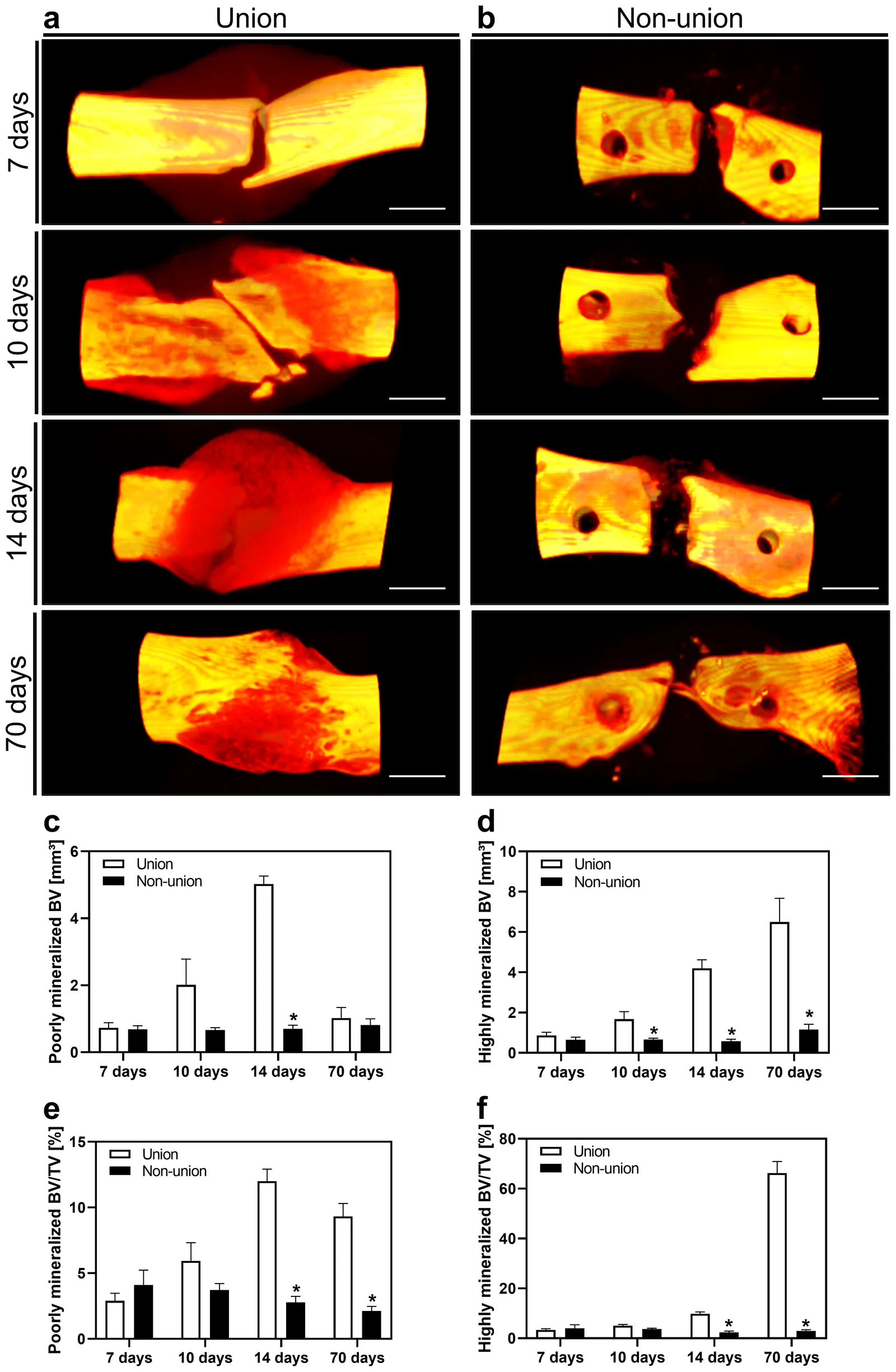
µCT. **(a, b)** Representative 3-dimensional µCT images of femora in the union **(a)** and non-union group **(b)** at 7, 10, 14 and 70 days after surgery. Scale bars: 1 mm. **(c, d)** µCT analysis of poorly **(c)** and highly mineralized **(d)** BV (mm³) in the union and non-union group at 7, 10, 14 and 70 days after surgery (n = 5 per group and time point). **(e, f)** µCT analysis of poorly **(e)** and highly mineralized **(f)** BV/TV (%) in the union and non-union group at 7, 10, 14 and 70 days after surgery (n = 5 per group and time point). Data are given as mean ± SEM; *p < 0.05 vs. union (Mann-Whitney U test).

Quantitative analyses of bone formation demonstrated a significantly lower poorly mineralized BV at 14 days and a significantly reduced highly mineralized BV in the non-union group at 10, 14 and 70 days (**Figures 2c, d**). Further analyses of the callus composition showed a significantly decreased poorly and highly mineralized BV fraction (BV/TV) within non-unions at 14 and 70 days after surgery (**Figures 2e, f**). Moreover, analyses of the trabecular architecture revealed a significantly increased trabecular thickness at 7 days in the non-union group whereas at 70 days the trabecular thickness was significantly decreased (**Table 1**). This was associated with a significantly higher trabecular separation at 70 days, whereas the trabecular number was significantly lower at 14 days after surgery in non-unions (**Table 1**).

**Table 1:** µCT analyses of the trabecular architecture of unions and non-unions (n = 5).

Group	7 days	10 days	14 days	70 days
Trabecular thickness (mm)				
Union	0.07 ± 0.00	0.13 ± 0.02	0.15 ± 0.01	0.38 ± 0.01
Non-union	0.13 ± 0.01*	0.14 ± 0.01	0.10 ± 0.01	0.09 ± 0.00*
Trabecular separation (mm)				
Union	0.76 ± 0.07	0.78 ± 0.08	0.57 ± 0.05	0.28 ± 0.02
Non-union	0.80 ± 0.13	0.78 ± 0.07	0.76 ± 0.08	0.83 ± 0.06*
Trabecular number (1/mm)				
Union	0.89 ± 0.16	0.98 ± 0.22	1.53 ± 0.14	1.00 ± 0.45
Non-union	0.62 ± 0.19	0.59 ± 0.13	0.55 ± 0.11*	0.54 ± 0.06

Data are given Mean ± SEM. *p < 0.05 versus Union (Mann-Whitney U test).

### Histology and histomorphometry

In the union group, Safranin-O stainings showed typical signs of endochondral fracture healing, indicated by abundant cartilage and subsequent bone tissue formation (**Figure 3a**). In the non-union group, the osteotomy gap was progressively filled with fibrous tissue until the end of the experiments. Confirming the radiological findings, no signs of successful bone regeneration were observed (**Figure 3b**).

**Figure 3:**
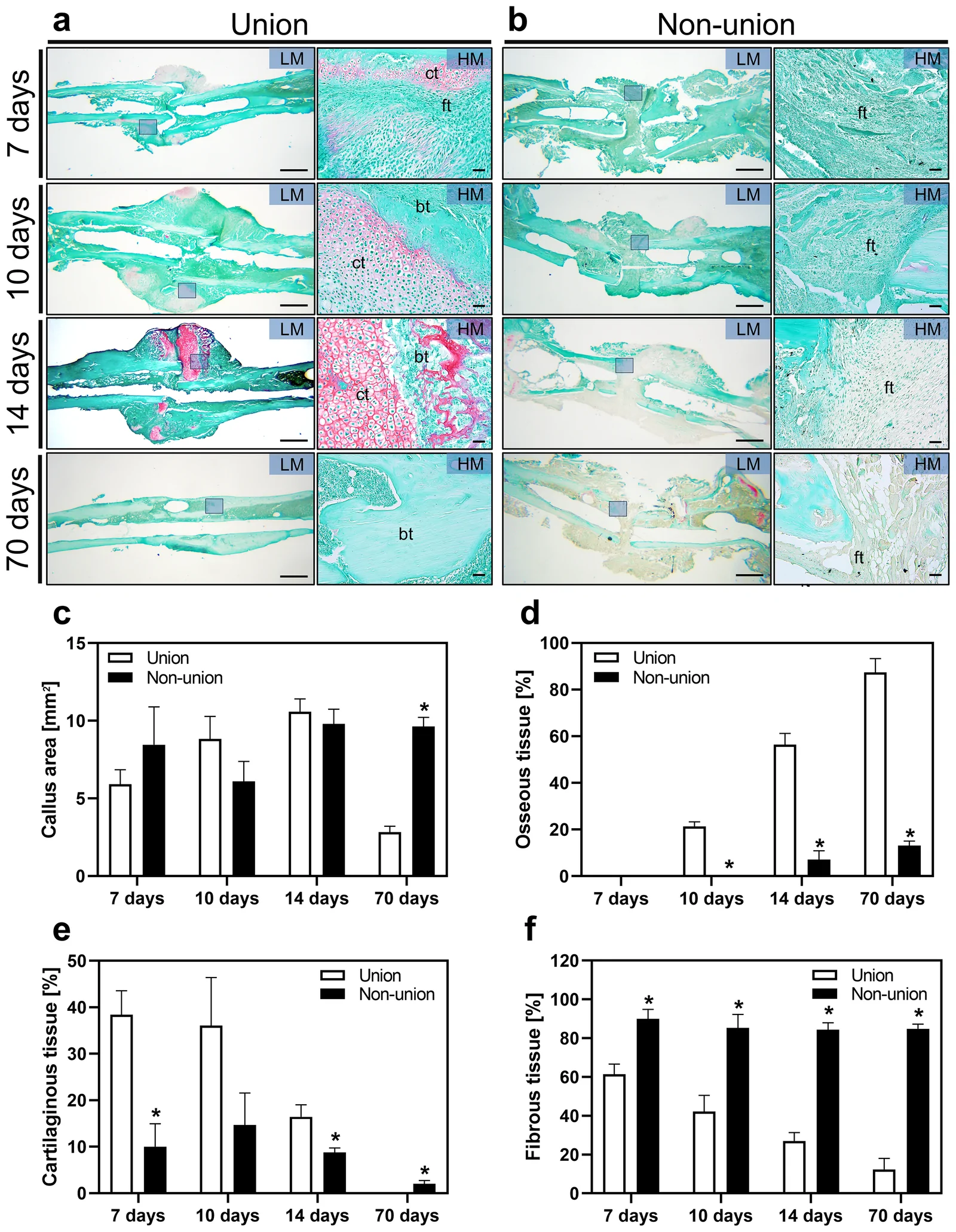
Histomorphometry. **(a, b)** Representative Safranin-O-stained sections (as low-magnification overview (LM, left panels) and high magnification of frames (HM, right panels) of femora in the union **(a)** and non-union group **(b)** at 7, 10, 14 and 70 days after surgery. Bone tissue (bt), cartilaginous tissue (ct) and fibrous tissue (ft) are indicated. LM scale bars: 1.0 mm. HM scale bars: 50 µm. (**c–f)** Histomorphometric analysis of the callus area [**(c)**, mm²], osseus tissue fraction [**(d)**, %], cartilaginous tissue fraction [**(e)**, %] and fibrous tissue fraction [**(f)**, %] in the union and non-union group at 7, 10, 14 and 70 days after surgery (n = 4–5 per group and time point). Data are given as mean ± SEM; *p < 0.05 vs. union (Mann-Whitney U test).

During the first 14 days the total callus area showed no significant differences between unions and non-unions (**Figure 3c**). However, at 70 days after surgery the histomorphometric analysis revealed a significantly larger total callus area of the femora in the non-union group when compared to the union group (**Figure 3c**). Further analyses of the callus composition demonstrated a significantly lower fraction of osseous tissue within the non-union group at 10, 14 and 70 days (**Figure 3d**). The ratio of cartilaginous tissue was also significantly reduced at 7 and 14 days after surgery in non-unions whereas it was significantly increased at 70 days (**Figure 3e**). As expected, the ratio of fibrous tissue was higher in the non-union group when compared to the union group throughout the entire observation period (**Figure 3f**).

### Immunohistochemistry

The infiltration of pro-inflammatory cells within the callus tissue of unions and non-unions was assessed by immunohistochemistry (**Figures 4a, b**). This analysis revealed an almost 3-fold higher number of MPO-positive cells at 10 days after surgery in the non-union group. In addition, no significant differences in the number of MPO-positive cells was detected at 7, 14 and 70 days after surgery between the two study groups (**Figures 4a, c**).

**Figure 4:**
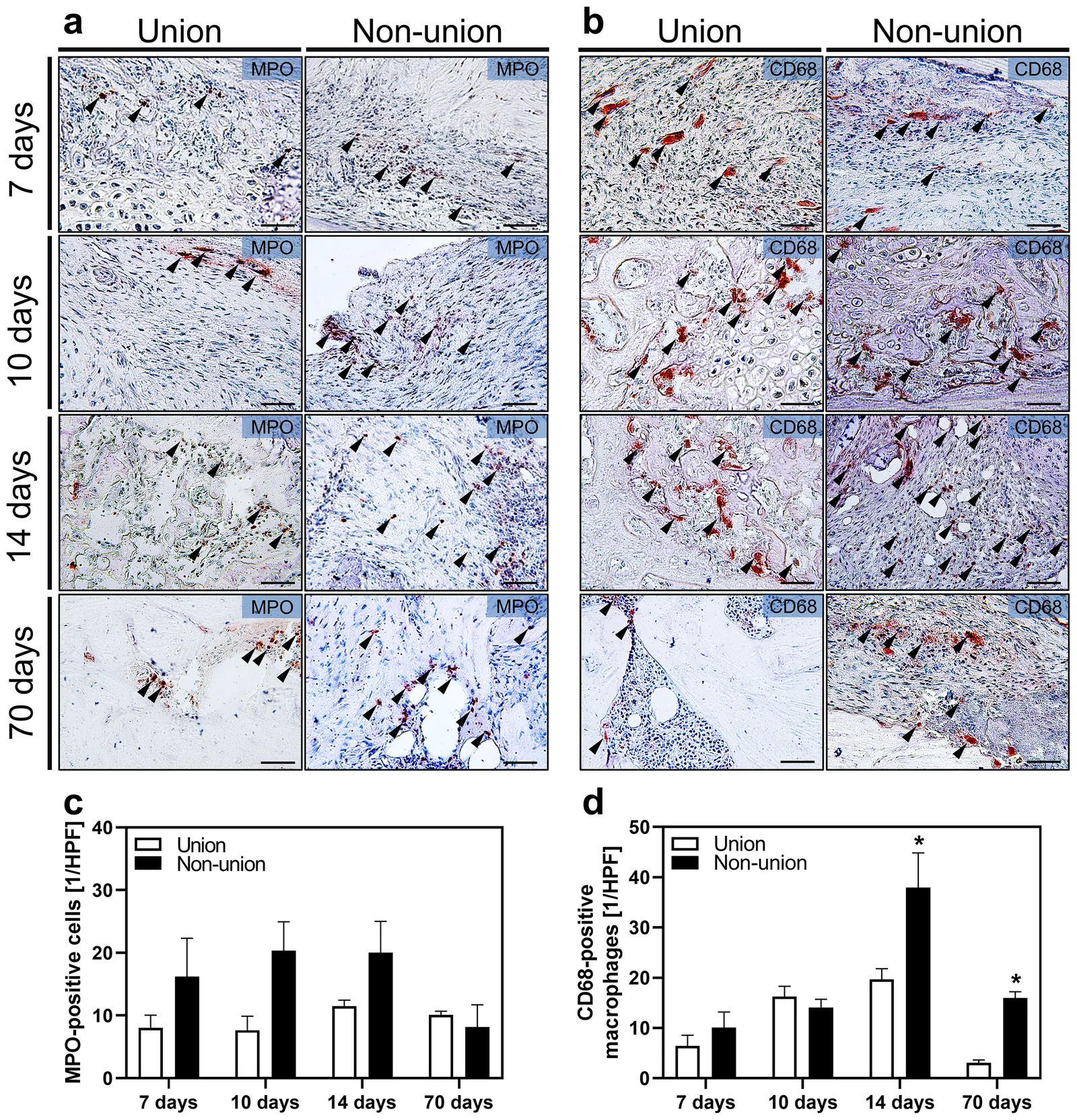
Inflammatory cell infiltration. **(a, b)** Representative immunohistochemical images of MPO-positive cells (a, arrowheads) and CD68-positive macrophages (b, arrowheads) in the union and non-union group at 7, 10, 14 and 70 days after surgery. Scale bars: 50 µm. **(c, d):** Immunohistochemical analysis of MPO-positive cells (1/HPF) **(c)** and CD68-positive macrophages (1/HPF) **(d)** in the union and non-union group at 7, 10, 14 and 70 days after surgery (n = 4–5 per group and time point). Data are given as mean ± SEM; *p < 0.05 vs. union (Mann-Whitney U test).

The number of CD68-positive macrophages was significantly higher in the non-union group at 14 and 70 days, whereas no significant differences were observed at the early healing time points 7 and 10 days after surgery (**Figures 4b, d**).

The additional immunohistochemical analysis within the callus tissue demonstrated a slightly higher number of p16-positive cells during the early healing period and at 70 days after surgery a significantly higher number in the non-union group when compared to unions (**Figures 5a, b**).

**Figure 5:**
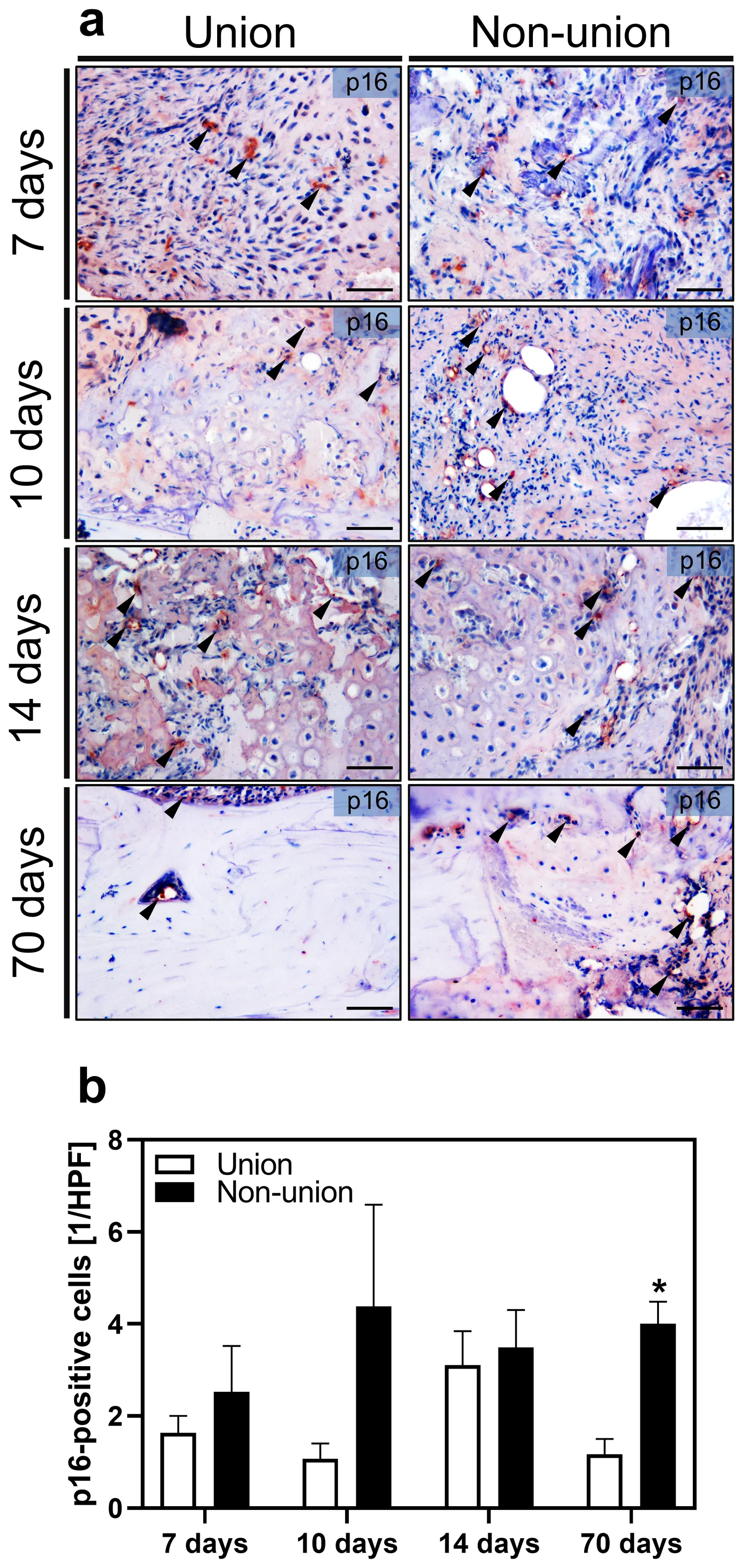
Cellular p16 expression. **(a)** Representative immunohistochemical images of p16-positive cells (arrowheads) in the union and non-union group at 7, 10, 14 and 70 days after surgery. Scale bars: 50 µm. **(b)** Immunohistochemical analysis of p16-positive cells (1/HPF) in the union and non-union group at 7, 10, 14 and 70 days after surgery (n = 4–5 per group and time point). Data are given as mean ± SEM; *p < 0.05 vs. union (Mann-Whitney U test).

### Western blot

The expression profile of inflammasomes within the callus tissue of unions and non-unions was analyzed by Western blotting. This analysis revealed a significantly higher expression of NLRP1 at 14 days and of NLRP3 at 10 days after surgery in the non-union group (**Figures 6a–c**). Moreover, the expression of NLRP3 showed a tendency towards a higher level at 7 and 14 days within non-unions (**Figures 6a–c**). In contrast, the expression of AIM2 was significantly lower in non-unions at 14 days after surgery when compared to unions (**Figures 6a, d**). The expression of the NLRC4 inflammasome showed no significant differences between the two study groups. However, a tendency towards a higher expression of NLRC4 in non-unions compared to unions could be observed at 14 days after surgery (**Figures 6a, e**).

**Figure 6:**
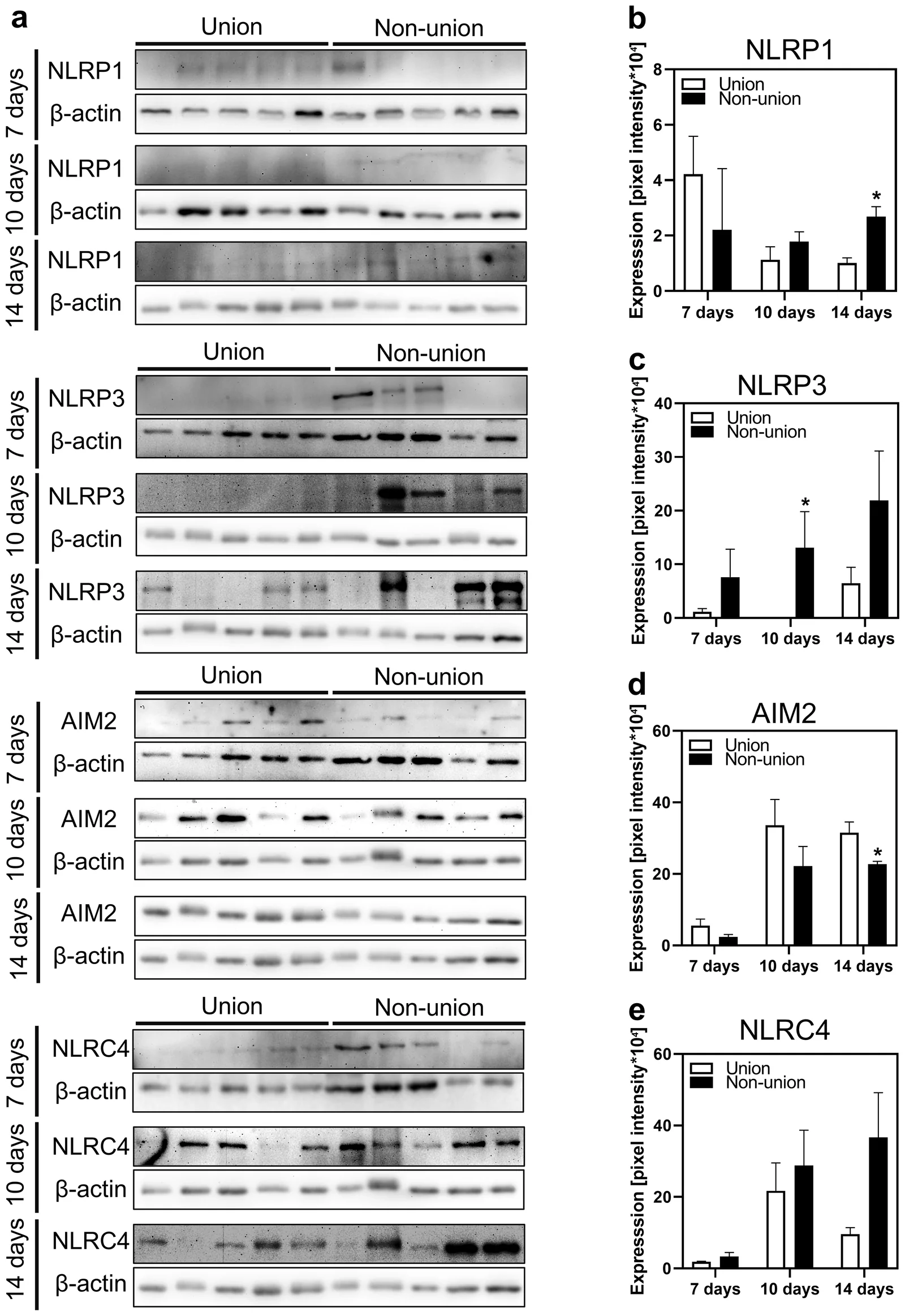
Inflammasome expression. **(a)** Western blots of NLRP1, NLRP3, AIM2, NLRC4 and β-actin expression within the callus tissue of the union and non-union group at 7, 10 and 14 days after surgery. **(b–e)** Western blot analysis of NLRP1 **(b)**, NLRP3 **(c)**, AIM2 **(d)** and NLRC4 **(e)** expression (pixel intensity*10^4^) within the callus tissue of the union and non-union group at 7, 10 and 14 days after surgery (n = 5 per group and time point). Data are given as mean ± SEM; *p < 0.05 vs. union (Mann-Whitney U test). β-actin loading controls are shared within and between **Figures 6** and **8**, as the following corresponding target proteins were detected on the same membranes: NLRP1 **(a)** and IL-1β (**Figure 8a**) at days 7, 10, and 14; NLRP3 **(a)**, AIM2 **(a)** and IL-18 (**Figure 8a**) at day 7; and NLRC4 **(a)**, AIM2 **(a)** and IL-18 (**Figure 8a**) at days 10 and 14).

Additional descriptive analyses of the ratios of mean inflammasome expression between the two study groups were performed to detect distinct expression signatures of successful fracture repair versus non-union formation. Interestingly, the NLRP3/NLRP1 and NLRP3/AIM2 ratios were higher within non-unions compared to unions throughout the healing process (**Figures 7a, b**). In addition, the NLRP3/NLRC4 ratio was increased at 7 and 10 days postoperatively in non-unions (**Figure 7c**). The NLRC4/AIM2 ratio was also higher in non-unions when compared to unions throughout the observation period, whereas the NLRC4/NLRP1 and NLRP1/AIM2 ratio showed a comparable course between the two study groups (**Figures 7d, e, f**).

**Figure 7:**
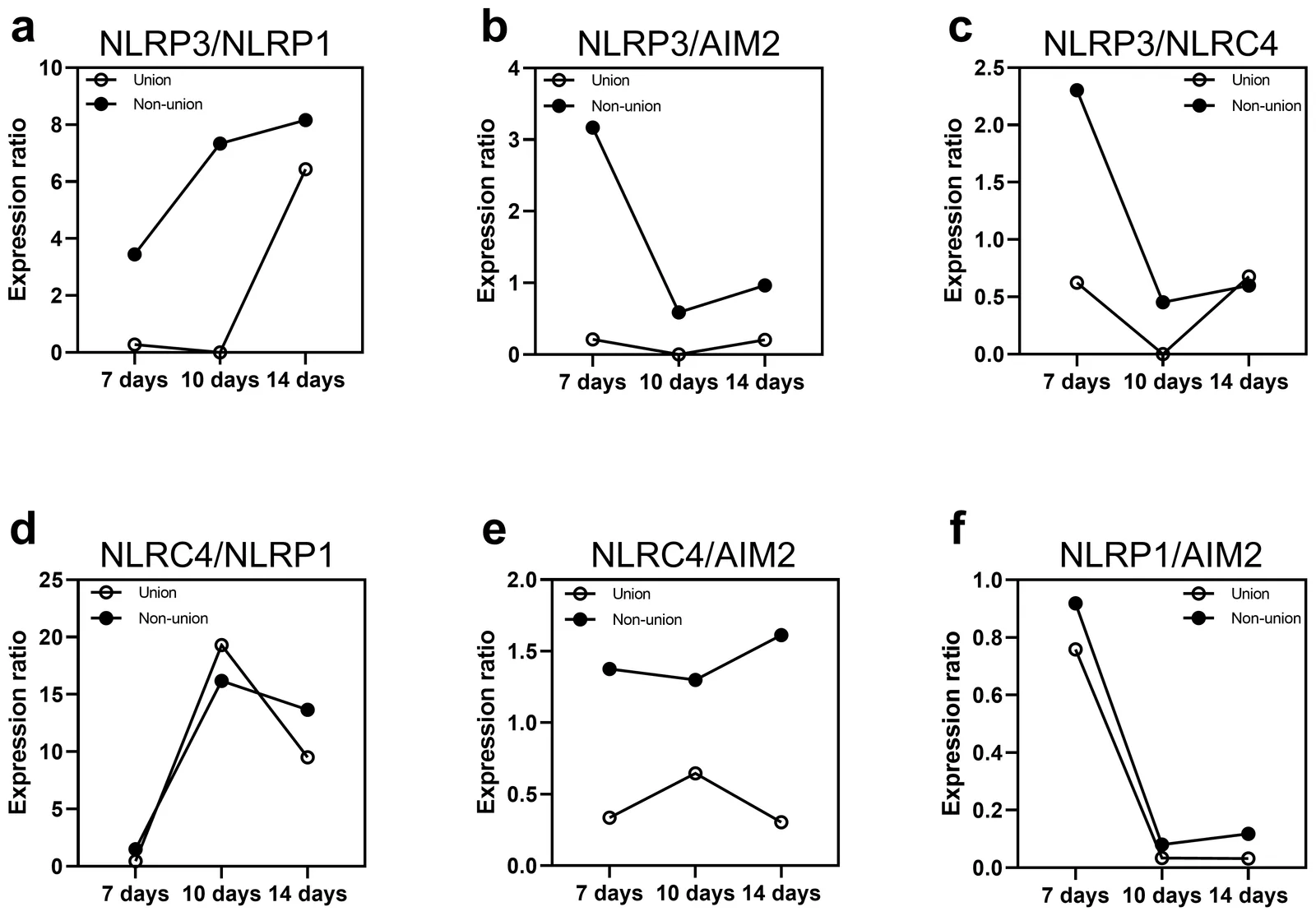
Inflammasome ratios. **(a–f)** Descriptive presentation of ratios of different inflammasome expressions in the union and non-union group at 7, 10 and 14 days after surgery. The following rations are indicated: NLRP3/NLRP1 **(a)**, NLRP3/AIM2 **(b)**, NLRP3/NLRC4 **(c)**, NLRC4/NLRP1 **(d)**, NLRC4/AIM2 **(e)** and NLRP1/AIM2 **(f)**. Data are given as ratio of means.

Finally, the Western blot analysis showed a clear trend towards a higher IL-1β expression within the callus tissue of non-unions at 14 days after surgery when compared to unions (**Figures 8a, b**). Of note, the expression of IL-18 did not significantly differ between the two study groups (**Figures 8a, c**).

**Figure 8:**
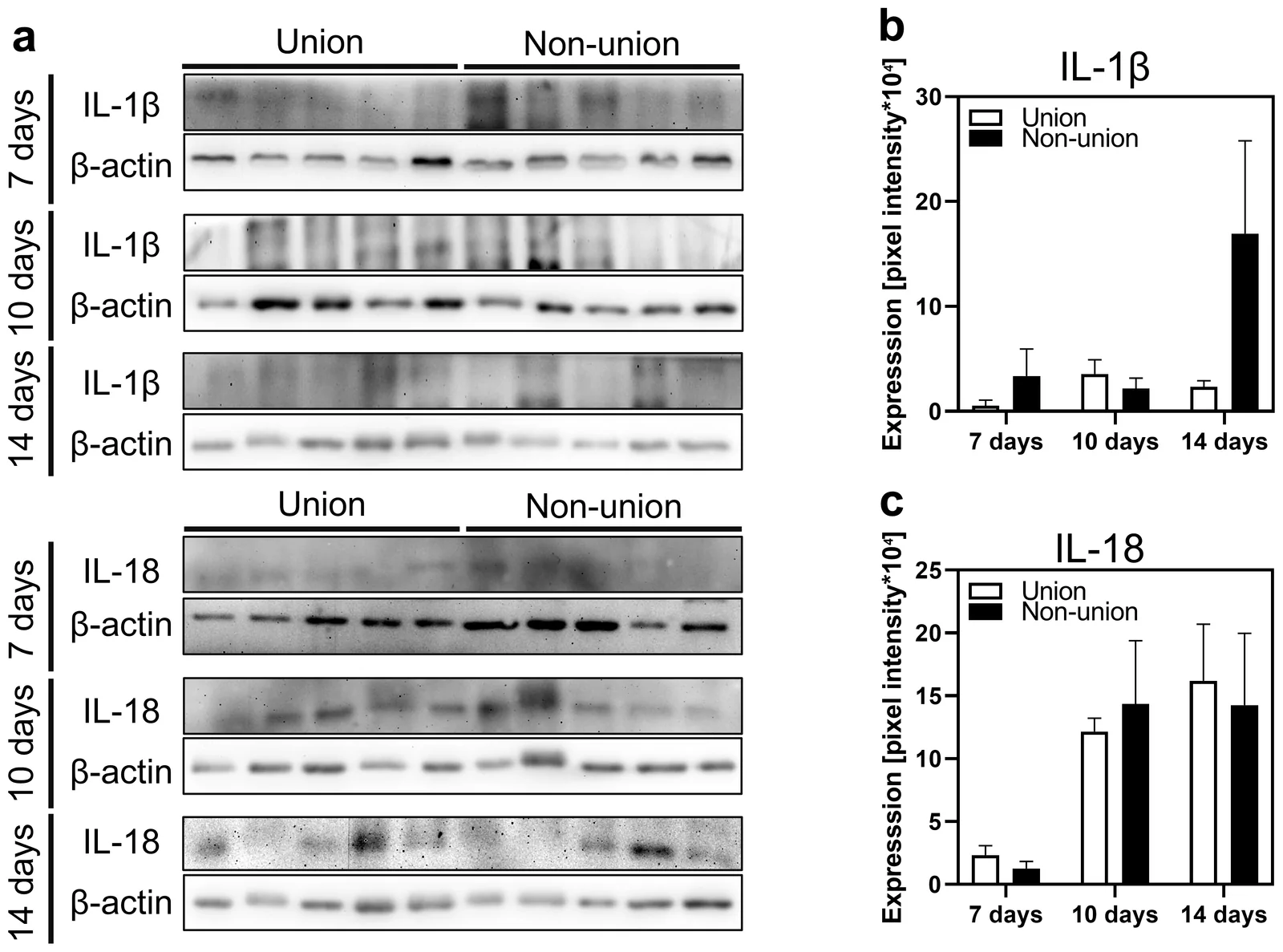
IL expression. **(a)** Western blots of IL-1β, IL-18 and β-actin expression within the callus tissue of the union and non-union group at 7, 10 and 14 days after surgery. Western blot analysis of IL-1β **(b)**, and IL-18 **(c)** expression (pixel intensity*10^4^) within the callus tissue of the union and non-union group at 7, 10 and 14 days after surgery (n = 5 per group and time point). Data are given as mean ± SEM; *p < 0.05 vs. union (Mann-Whitney U test). β-actin loading controls are shared within and between **Figures 6** and **8**, as the following corresponding target proteins were detected on the same membranes: NLRP1 (**Figure 6a**) and IL-1β **(a)** at days 7, 10, and 14; NLRP3 (**Figure 6a**), AIM2 (**Figure 6a**) and IL-18 **(a)** at day 7; and NLRC4 (**Figure 6a**), AIM2 (**Figure 6a**) and IL-18 **(a)** at days 10 and 14).

## Discussion

Inflammatory dysregulation at the fracture site significantly contributes to delayed bone healing and non-union formation (21). In light of this fact, the present study characterized and compared for the first time the expression profiles of different inflammasomes during successful fracture healing and non-union formation. Our results showed a higher expression of the NLRP1 and NLRP3 inflammasome within the callus tissue of non-unions, whereas the expression of the AIM2 inflammasome was significantly lower when compared to unions. This altered inflammasome expression was associated with an increased infiltration of pro-inflammatory MPO-positive cells and macrophages as well as a higher number of p16-positive cells within the callus tissue of non-unions.

For the investigation of the cellular and molecular basis of facture healing, murine models have gained increasing interest in the last decades. Fracture models in mice enable a thorough cellular and biochemical analysis of the callus tissue by a wide array of mono- and polyclonal antibodies (22). This allows a detailed analysis of pro-inflammatory cells and cytokines at the fracture site. In the present study, we used a standardized fracture model in mice using a 3-point bending device and an intramedullary compression screw (14) to mimic the process of successful fracture healing (union group). Radiological and histomorphometric analyses showed typical patterns of endochondral fracture healing during the early healing phase and complete bone remodeling 70 days after surgery. For the induction of non-union formation we used the well-established pin-clip model by Garcia et al. (15). This approach resulted in an atrophic non-union rate of 100%, as indicated at 70 days after surgery by the lack of bone formation, rounded bone fragments and abundant fibrous tissue within the fracture gap. Of note, 70 days was chosen as the final observation time point, as it represents the minimum period without healing to define non-union formation in mice (15).

The inflammatory response to injury is a critical prerequisite for successful fracture repair. Inflammatory cytokines secreted by granulocytes and macrophages, such as tumor necrosis factor (TNF)-α and IL-6, create a microenvironment that is vital for the onset of callus and subsequent bone formation (5, 23). On the other hand failure to resolve this initial inflammatory response, may have detrimental effects on bone regeneration (21). In fact, there is evidence that an overshooting and prolonged inflammatory state mediated by pro-inflammatory cytokines and cells can result in decreased mineralization and bone formation associated with impaired vascularization and a decreased bone turnover by osteoclast and osteoblasts (24, 25). In line with these findings, we could demonstrate that the inflammatory cell pattern within the callus tissue of non-unions is characterized by increased numbers of MPO-positive cells and macrophages at an early stage of healing failure, with the latter remaining significantly elevated at the end of non-union formation.

This overshooting and prolonged inflammatory state may be mediated by an altered inflammasome expression within the callus tissue of non-unions. The NLRP3 inflammasome is one of the best characterized inflammasomes to date. Interestingly, there is evidence that NLRP3 is associated with impaired bone regeneration. Wang et al. (26) demonstrated by the pro-inflammatory stimulation of human MSCs with lipopolysaccharide and palmitic acid that NLRP3 inhibits their pro-osteogenic differentiation. Moreover, NLRP3 suppression and deficiency reduce alveolar bone loss and promote bone healing under pro-inflammatory conditions, such as periodontitis (27) and diabetes (28). In addition, inhibiting the expression of NLRP3 reduces bone loss associated with age-related osteoporosis (29). In line with these findings, our Western blot analyses revealed a higher NLRP3 expression within the callus tissue of non-unions when compared to unions, indicating the detrimental effects of NLRP3 on fracture repair. Notably, within the callus tissue of unions, NLRP3 showed only a low expression. Moreover, in a previous study we could demonstrate that *Nlrp3* deficiency has only marginal effects on the process of fracture healing (30). Thus, we conclude that while excessive NLRP3 expression may contribute to fracture healing failure, its absence does not significantly affect the outcome of fracture repair.

Interestingly, our analyses also showed a significantly higher expression of the NLRP1 inflammasome during non-union formation. To our knowledge there is only little information on the effects of NLRP1 on bone metabolism and fracture healing. Hong et al. (31) could demonstrate that a NLRP1 knockout during bone marrow transplantation reduces the inflammatory response and promotes hematopoietic reconstitution. However, at the same time the NLRP1 knockout decreased the osteogenic differentiation of MSCs (31). These conflicting results may be explained in the way that both an increased NLRP1 expression as well as a complete abrogation of NLRP1 expression, may contribute to inflammatory dysregulation, thereby hindering adequate fracture repair. In contrast, a basal NLRP1 expression may be necessary to induce an appropriate inflammatory response, resulting in successful fracture healing.

In contrast to the increased NLRP1 and NLRP3 expression, we found a reduced expression of the AIM2 inflammasome in the non-union group. Of note, Gong et al. (13) reported that AIM2-null mice exhibit a reduced BMD within the distal femur metaphysis compared to age-matched wildtype control mice. This is associated with a shift towards the pro-adipogenic pathway of pro-osteogenic progenitor cells within the bone marrow of AIM2-null mice (13). Hence, AIM2 may directly contribute to osteogenesis by promoting the differentiation of MSCs towards a pro-osteogenic phenotype. Hence, the reduced AIM2 expression within the callus tissue of non-unions may have contributed to fracture healing failure. Notably, AIM2, NLRP1 and NLRP3 have similar downstream targets, such as IL-1β and IL-18 (10, 32). Therefore, the reduced AIM2 expression in non-unions may explain why our Western blot analysis only showed a tendency towards an increased IL-1β expression, and no significant difference in IL-18 expression. However, the pro-inflammatory mechanisms of the different inflammasome subtypes are highly complex and not yet fully understood. Potential inflammasome-dependent mechanisms and mediators contributing to the observed pro-inflammatory cell infiltration within the callus tissue of non-unions include pyroptosis (10), lipid mediators such as eicosanoids (33), and nuclear factor (NF)κB (34). Future studies are needed to further elucidate the distinct pro-inflammatory pathways of the different inflammasome subtypes that contribute to the pro-inflammatory cell migration during successful fracture healing versus non-union formation.

Furthermore, the various inflammasomes can influence each other’s function (10). For example, NLRC4 has a modulatory effect on NLRP3 expression during bone remodeling (35). Notably, in the present study NLRC4 expression in non-unions tended to be up-regulated 14 days after surgery when compared to unions. This was associated with a markedly higher NLRC4/AIM2 ratio within the callus tissue of non-unions when compared to the union group. However, the exact mechanisms on how NLRC4 may influence the healing outcome remain elusive. Still, in addition to NLRP1, NLRP3 and AIM2, NLRC4 may represent a promising marker in callus tissue samples obtained during revision surgery, potentially predicting the healing outcome in patients suffering from compromised fracture healing.

The protein p16 is well established as regulator of cell cycle progression by inhibiting the S phase, which is frequently upregulated during cellular stress (36). Saul et al. (37) could demonstrate that p16 is transiently expressed within the fracture callus of mice and that the pharmacological inhibition of p16-positive cells accelerates the process of bone regeneration (37). In line with these findings, we found a significantly increased number of p16-positive cells within the callus tissue of non-unions when compared to unions at the end of the healing process. It may be speculated that although p16 expression is necessary during phycological fracture repair, its persisting accumulation may hinder adequate bone regeneration and result in healing failure.

Moreover, p16 is an important marker for the indication of cellular senescence (38). Senescent cells induce a deleterious pro-inflammatory environment for bone tissue and is associated with age-related bone loss (39, 40). Treatment approaches inhibiting cellular senescence may be a promising strategy to overcome bone loss and even improve the outcome of fracture healing (37, 41, 42). In this context, the NLRP1 inflammasome is known to act as a mediator for the senescence-associated secretory phenotype (SASP) (43). Thus, it may represent a promising target for stimulating bone regeneration.

Of note, several limitations of this study should be acknowledged. Although MPO is mainly expressed within neutrophilic granulocytes, it can also be detected in other myeloid cell populations, including monocytes and macrophages, making it an unspecific cell marker (44, 45). Future studies should include additional lineage-specific markers, such as Ly6G for neutrophilic granulocytes to enable a more precise characterization of the infiltrating immune cell populations. Furthermore, the sole expression of p16 is insufficient for confirming cellular senescence. Additional senescence-associated markers, such as p21 and components of the senescence-associated secretory phenotype (SASP), would be required to validate a senescent phenotype (46, 47). Moreover, double-immunostaining, which specifically characterize the p16- and NLRP3 positive cell population (such as granulocytes or macrophages) is necessary to fully elucidate the interplay of pro-inflammatory cells, senescence and inflammasome expression within the process of fracture repair. Another limitation of the present study is the lack of mechanistic and therapeutic approaches. Although our data demonstrate an association between altered inflammasome expression and non-union formation, they do not establish a causal relationship between inflammasome activation and impaired fracture healing. In addition, due to the limited number of animals and the use of a single mouse strain, the potential influence of sex and genetic background on inflammasome expression could not be evaluated. Moreover, the assessment of additional inflammasome activation markers, such as cleaved caspase-1 and ASC speck formation, would provide more direct evidence of inflammasome activation and help elucidate the underlying mechanisms. Therefore, future studies employing both genetic and pharmacological approaches to inhibit or activate inflammasome signaling are warranted to determine the functional role of inflammasome activation in fracture healing and to evaluate its potential as a therapeutic target.

## Conclusion

Taken together, the present study characterized and compared for the first time the distinct expression profiles of inflammasomes during successful fracture healing and non-union formation. Our novel findings suggest that specific inflammasome signatures within the callus tissue may serve as predictive biomarkers to assess the likelihood of fracture healing success or failure after revision surgery. Furthermore, modulating inflammasome expression may represent a novel therapeutic approach to enhance bone regeneration and prevent non-union formation in patients at risk for impaired fracture healing.

## Data Availability

The raw data supporting the conclusions of this article will be made available by the authors upon reasonable request.
